# Physiological evaluation of free-ranging moose (*Alces alces*) immobilized with etorphine-xylazine-acepromazine in Northern Sweden

**DOI:** 10.1186/1751-0147-54-77

**Published:** 2012-12-31

**Authors:** Alina L Evans, Åsa Fahlman, Göran Ericsson, Henning Andreas Haga, Jon M Arnemo

**Affiliations:** 1 Department of Forestry and Wildlife Management, Hedmark University College, Campus Evenstad, Koppang, NO-2480, Norway; 2 Section of Arctic Veterinary Medicine, Norwegian School of Veterinary Science, Tromsø, NO-9292, Norway; 3 Faculty of Veterinary Medicine and Animal Science, Swedish University of Agricultural Sciences, Uppsala, SE-750 07, Sweden; 4 Faculty of Veterinary Medicine, University of Calgary, Calgary, Alberta, T2N 4N1, Canada; 5 Department of Wildlife, Fish and Environmental Studies, Swedish University of Agricultural Sciences, Umeå, SE-901 83, Sweden; 6 Department of Clinical Sciences, Norwegian School of Veterinary Science, Oslo, NO-0033, Norway

**Keywords:** Alces, Moose, Anesthesia, Etorphine, Xylazine, Immobilization

## Abstract

**Background:**

Evaluation of physiology during capture and anesthesia of free-ranging wildlife is useful for determining the effect that capture methods have on both ecological research results and animal welfare. This study evaluates capture and anesthesia of moose (*Alces alces*) with etorphine-xylazine-acepromazine in Northern Sweden.

**Methods:**

Fifteen adult moose aged 3–15 years were darted from a helicopter with a combination of 3.37 mg etorphine, 75 mg xylazine, and 15 mg acepromazine. Paired arterial blood samples were collected 15 minutes apart with the first sample at 15–23 minutes after darting and were analyzed immediately with an i-STAT®1 Portable Clinical Analyzer.

**Results:**

All animals developed hypoxemia (PaO_2_ <10 kPa) with nine animals having marked hypoxemia (PaO_2_ 5.5-8 kPa). All moose were acidemic (ph<7.35) with nine moose having marked acidemia (pH<7.20). For PaCO_2,_ 14 moose had mild hypercapnia (PaCO_2_ 6-8 kPa) and two had marked hypercapnia (PaCO_2_>8 kPa). Pulse, respiratory rate, pH and HCO_3_ increased significantly over time from darting whereas lactate decreased.

**Conclusions:**

The hypoxemia found in this study is a strong indication for investigating alternative drug doses or combinations or treatment with supplemental oxygen.

## Background

Potent opioids (etorphine, carfentanil or thiafentanil) are considered the drugs of choice for immobilization of free-ranging moose [1-3]. In Sweden, 1,263 moose captures were carried out with etorphine-acepromazine-xylazine from a helicopter 1984–2003, with an overall mortality rate of 1.0% [4]. During the same period, 1,491 captures of moose were done in Norway with etorphine, with an overall mortality rate of 0.5% [4]. Other opioids and combinations of opioids used in moose include carfentanil-xylazine [5,6], carfentanil [6] and thiafentanil [3]. In other studies of different subpopulations of moose, xylazine and carfentanil in combination resulted in much higher mortality rates [4-6]. A study of moose captures using thiafentanil [3] reported induction and recovery times of less than 4 minutes, but the mortality rate was 5% within the first 30 days of capture with causes of death including pneumonia, malnutrition and undetermined

While low mortality rates are important, they cannot stand alone as the measure of capture success and immobilization safety. Evaluation of capture physiology has both research and welfare implications. Altered physiology during capture can affect research results including movement and activity levels [7]. No effect on calving rates of captured females was documented when a combination of carfentanil, fentanyl, xylazine and hyaluronidase was used [8]. However, calves born to females captured in the last 3 months of pregnancy had lowered postnatal survivorship than those born to females not captured during this period [8]. A recent study evaluating movement pre and post capture with etorphine-acepromazine-xylazine found abnormally increased movements for up to 4.5 days after capture with no further residual effect [9].

Arterial blood gases and acid-base status are valuable for evaluating capture methods and their physiological effects. Acidosis and hypoxemia could have many undesirable and undetected effects including organ dysfunction [10] and damage to the brain [11], liver [12,13] and kidneys [14], and in sheep, has been shown to result in fetal hypoxemia [15], leading to fetal brain damage [16]. These effects are difficult to evaluate in wildlife but may have consequences for the quality of results of the ecological studies dependent on capturing representative animals.

To our knowledge, physiological evaluation of immobilized moose including blood gases and acid–base status has not been reported previously. The aim of this study was to evaluate the physiology, including vital signs and blood oxygen and acid–base status, of moose immobilized with etorphine-acepromazine-xylazine.

## Methods

The study was conducted during March 2010 in Nikkaluokta, Sweden (67°52’ N, 18°66’ E). Twelve female and three male moose three to fifteen years old were captured and collared as part of ongoing research. All captures were approved by the Ethical Committee on Animal Experiments in Umeå, Sweden. All moose had been captured and equipped with radiocollars and/or GPS collars three years previously. The moose were aged based on tooth wear (calibrated with sectioning [17]) during immobilizations one year prior to these captures. Animals were not weighed.

The moose were located based on GPS positions and radio tracking. A CO_2_ powered rifle (Dan-Inject, Børkop, Denmark) was used to deliver a dart injection in the gluteal or epaxial muscles from less than 10 m away by helicopter. The dart contained a mixture of 75 mg xylazine (Rompun®, Bayer AG, Leverkusen D-51368, Germany), 3.37 mg etorphine and 15.0 mg acepromazine (Large Animal Immobilon®, Novartis Animal Health, Litlington, UK, 2.25 mg/ml etorphine and 10 mg/ml acepromazine). Moose not recumbent after 15 minutes were given a second dart with 2/3rds of the original dose. All darts had a Recco tracking device (Recco AB, Lidingö, Sweden) to ensure that they could be recovered.

Altitude was determined by correlating GPS points recorded from the helicopter with a detailed terrain map. Snow depth was measured in three places along the final 15 meters that the moose walked with a meter stick and the average was used. Ambient temperature and barometric pressure were also recorded.

Recorded variables included time from sighting animal to successful darting (darting time), time from darting to recumbency (induction time), and estimated distance run (distance from initial sighting to recumbency). All moose were maintained in sternal recumbency during immobilization. Pulse rate (by palpation of auricular artery), respiratory rate (counting thoracic excursions), and rectal temperature (using a digital thermometer) were measured as soon as possible after recumbency (upon capture) and repeated 15 minutes later. On capture and approximately 15 minutes later, arterial blood samples were collected anaerobically from the auricular artery using self-filling arterial syringes with heparin (PICO™ 70, Radiometer Copenhagen, Brønshøj, Denmark) and analyzed immediately with an i-STAT®1 Portable Clinical Analyzer and i-STAT® CG4+ and EC8+ cartridges (Abbott Laboratories, Abbott Park, Illinois 60064–6048, USA). The i-STAT®1 analyzer was kept in an insulated box to keep it at optimum temperature (16-30°C), with warm water bottles used as needed. Measured variables included pH, hematocrit, partial pressure of arterial oxygen (PaO_2_) and carbon dioxide (PaCO_2_) and whole blood concentrations of lactate, sodium (Na), potassium (K), chloride (Cl), and glucose. PaO_2_, PaCO_2_ and pH were corrected based on rectal temperature. Calculated values included concentration of bicarbonate (HCO_3_), hemoglobin, oxygen hemoglobin saturation (SaO_2_%), base excess (BE), and anion gap (AG).

Etorphine was reversed with diprenorphine (Large Animal Revivon® 3 mg/ml, Novartis Animal Health) at a dose ratio at 1.33 mg per mg etorphine. Xylazine was reversed with a ratio of 0.1 mg atipamezole per mg xylazine (Antisedan®, 5 mg/ml, Orion Pharma Animal Health, Turku, Finland) [18]. Time from darting until time of administration of reversal drugs and time from reversal until standing were recorded.

Hypoxemia was defined as mild (PaO_2_ 8.0-10.0 kPa), marked (PaO_2_ 5.5-8.0 kPa), or severe (PaO_2_ <5.5 kPa). Acidemia was defined as a pH <7.35, and acidemia was considered marked if pH <7.20. Hypocapnia was defined as a PaCO_2_ <4.5 kPa and hypercapnia was defined as mild (PaCO_2_ 6–8 kPa) or marked (PaCO_2_ >8 kPa).

The alveolar oxygen tension (PAO_2_) was calculated based on the alveolar gas equation *PAO*_2_ = *FiO*_2_(*PB* − *P*_*H*20_) − (*PaCO*_2_/*RQ*)]. FiO_2_ = fraction of inspired oxygen (0.21). P_H2O_ = saturated vapor pressure for water at 37°C (47 mmHg). Barometric pressure (PB) was recorded with the iSTAT and 1.0 was used for the respiratory quotient (RQ) [19]. The aveolar-arterial oxygen tension difference [P(A-a)O_2_ was calculated by subtracting PaO_2_ from PAO_2_.

### Statistics

Variables were first tested for normality using Kolmogorov-Smirnov test for normality (p<0.05). Results for the first and second sample (both measured and calculated values) were compared using Wilcoxon signed rank test. Statistics were carried out using JMP® 9.0.2 (SAS Institute, NC, USA).

## Results

**Figure 1:**
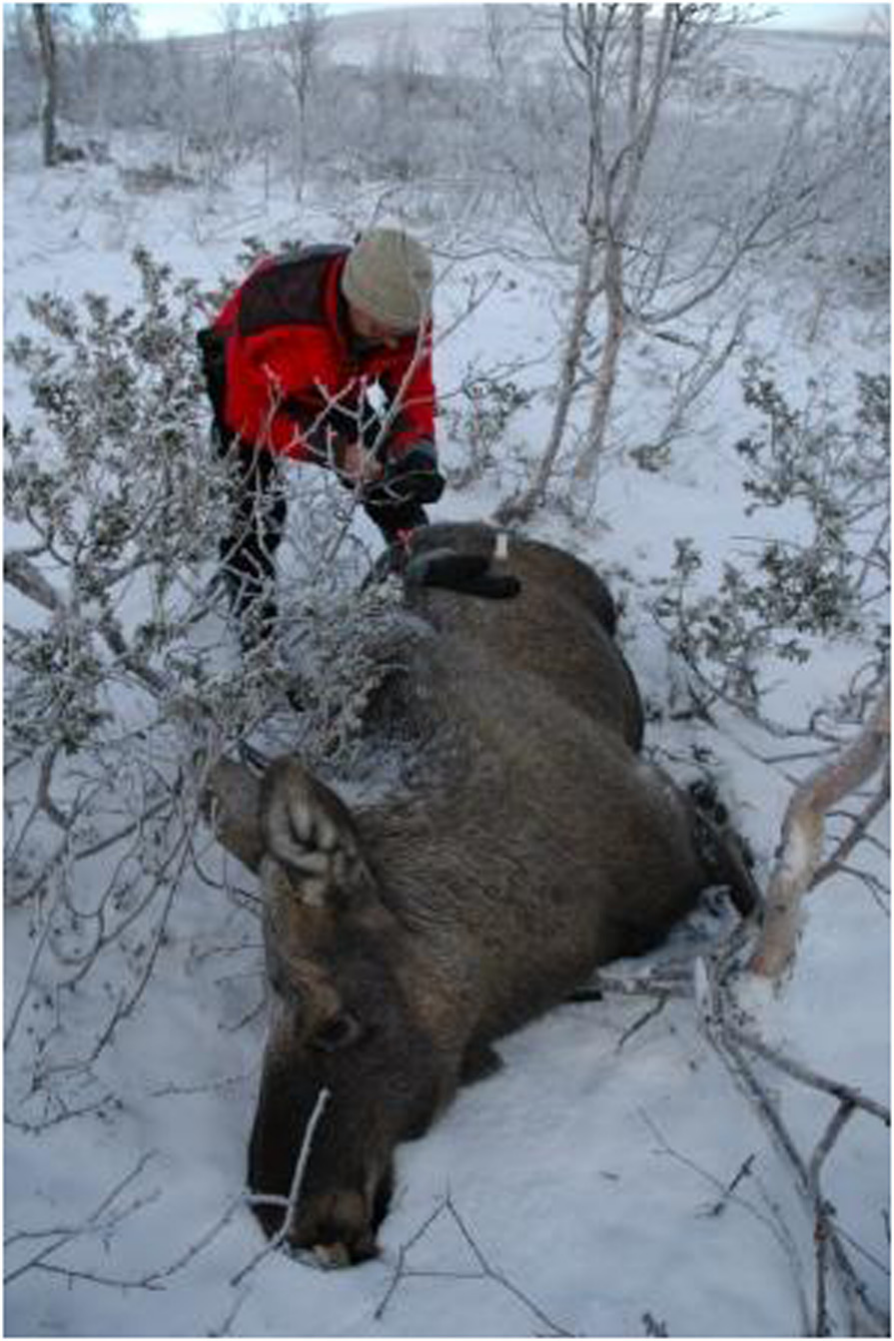
**Moose with head in the snow.** This moose, immobilized with the combination of etorphine-acepromazine-xylazine, exhibits open eyes with the pupils positioned centrally and the head lowered into the snow.

Moose were either in sternal recumbency on initial approach, or placed in sternal recumbency before sampling. The immobilization in general was characterized by open eyes, pupils positioned centrally and the head lowered, often into the snow, in which case the snow was cleared from the area in front of their nostrils (Figure 1).

Mean ± SD (range) of ambient temperature was −8 ± 3 (−4 to −15) degrees Celsius and snow depth was 52 ± 15 (30–75) cm. Altitude ranged from 444 to 595 meters above sea level with a barometric pressure from 694 to 708 mmHg. The initial doses used in this study were sufficient to immobilize 12/15 (80%) of moose on the first dart. Recumbency occurred in 6.5 ± 2.5 (3.5-11.5) minutes except for three animals that required a second dart 16–21 minutes after darting. These three animals were recumbent in 2, 3 and 9 minutes after the second dart. Between discovery and the first dart, moose moved an estimated 428 ± 303 (20–1,100) meters in 3.5 ± 3 (1–13) minutes. Once recumbent, no animals required supplemental drug doses. Atipamezole and diprenorphine were administered intravenously at 43 ± 10 (30–66) minutes after last darting. Mean time to standing was 2:12 ± 0:28 (1:30–3:20) min:sec.

Physiological variables and arterial blood gas results for moose with paired samples are presented in Table 1. All variables were normally distributed except for rectal temperature, and SaO_2_. All 15 moose had at least one PaO_2_ measurement below 10 kPa (mild hypoxemia) and 9 moose were between 5.5-8.0 kPa (marked hypoxemia) with two moose exhibiting severe hypoxemia (<5.5 kPa). For PaCO_2,_ 14 moose had at least one measurement above 6kPa (mild hypercapnia) and two had at least one measurement above 8 kPa (marked hypercapnia). PaO_2_ did not significantly change between sample 1 and 2 (Table 1). All moose were acidemic (pH< 7.35) and the acidemia was classified as marked in 9/15 moose (pH<7.20). The PAO_2_ and P(A-a)O_2_ did not differ between the first and second samples. PAO_2_ was 11.3 ± 1.0 (9.8-13.2) kPa between 15–23 minutes and 11.3 ± 1.0 (9.8-13.2) kPa from 29–43 minutes. P(A-a)O_2_ was 3.0 ± 1.4 (0.7-5.6) kPa between 15–23 minutes and 3.3 ± 1.2 (1.1-4.9) kPa from 29–43 minutes.

**Table 1:** Physiological variables during anesthesia of moose

**Time from darting**			**T1: 15–23 minutes**	**T2: 29–43 minutes**	
**Variable**	**Units**	**N**	**Mean±SD (Range)**	**Mean±SD (Range)**	**Z**
Pulse Rate	beats/min	10	46±8 (32–60)	45±8 (36–60)	*NSD*
Repiratory Rate	breaths/min	10	39±7 (28–52)	34±9 (22–48)	*NSD*
Rectal Temp^#^	C	10	37.7±0.5 (37.2-38.7)	37.4±0.6 (36.6-38.7)	*NSD*
PaO_2_^*^	kPa	10	8.46±1.90 (5.2-10.8)	8.12±1.94 (4.8-11)	*NSD*
	mmHg	10	63.5±14.2 (39.0-81.0)	60.9±14.6 (36.0-82.5)	*NSD*
PaCO_2_^*^	kPa	10	6.98±1.12 (5.29-8.63)	7.21±1.14 (5.76-9.31)	*NSD*
	mmHg	10	52.4±8.4 (39.7-64.7)	54.1±5.9 (43.2-69.8)	*NSD*
SaO_2_	%	10	79±13 (70–89)	79±20 (49–95)	*NSD*
pH^*,†^		10	7.15±0.12 (6.95-7.35)	7.21±0.10 (7.03-7.35)	0.0002
PAO_2_	kPa	10	11.3±1.0 (9.8-13.2)	11.3±1.0(9.8-13.2)	
	mmHg		84.9 ± 7.4 (73.7-99.1)	85.1 ± 7.4 (73.7-99.1)	*NSD*
P(A-a)O_2_	kPa	10	3.0±1.4 (0.7-5.6)	3.3±1.2 (1.1-4.9)	
	mmHg		22.2 ± 10.2 (5.3-42.1)	24.8 ±8.8 (8.1-36.8)	*NSD*
Lactate^†^	mmol/l	10	12.6±5.1 (5.0-19.8)	9.8±4.7 (2.8-16.4)	0.0002
Anion Gap^†^	mEq/l	9	20.9±3.9 (16–26)	18.6±4.2 (12–25)	0.0059
Na	mmol/l	9	137.6±1.0 (136–139)	136.8±1.6 (135–140)	*NSD*
K^†^	mmol/l	9	3.5±0.3 (2.9-3.8)	3.7±0.3 (3.2-4.1)	0.0391
Cl	mmol/l	9	101.9±2.0 (99–105)	101.8±3.1 (98–108)	*NSD*
Glucose	mmol/l	9	6.6±0.6 (5.7-7.6)	7.0±1.0 (5.6-8.5)	*NSD*
Hct^†^	% PCV	9	41.6±4.2 (33–46)	38.6±3.2 (33.0-42.0)	0.0063
Hb^†^	g/L	9	141±14 (112–143)	131±11 (112–143)	0.0059
BE^†^	mmol/l	10	−10.8±5.8 (−18-(−1))	−6.4±5.2 (−13-(+3))	0.0002
HCO3^†^	mmol/l	10	18.0±4.0 (13.4-24.5)	21.5±4.0 (17.0-28.9)	0.0002

*Blood gas and pH values were corrected to rectal temperature.

Mean and standard deviation of physiological variables at 15–23 and 29–43 minutes for moose darted once. SaO_2_=hemoglobin oxygen saturation. PaO_2_ and PaCO_2_ = partial pressures of arterial oxygen and carbon dioxide.

All moose were living seven months after capture except for one male, which was harvested during the regular hunting season six months after capture.

## Discussion

This study of 15 animals presents physiological variables in moose anesthetized with etorphine-acepromazine-xylazine. To the best of our knowledge, this type of evaluation has not previously been reported for anesthetic protocols in moose. This study documented hypoxemia, hypercapnia and acidemia in moose immobilized with this combination.

Although the mean induction time for the 12 moose immobilized with one dart in our study was acceptable, it was over 2 minutes longer than the 4.4 ± 2.6 minutes reported in etorphine-immobilized moose [20]. Pulse and rectal temperature did not change over time and were within acceptable ranges for this species. Guidelines for cervid anesthesia include taking corrective measures when a rectal temperature over 40°C or a heart rate under 30 beats per minute is observed [2].

Although the moose were kept in sternal recumbency and snow was cleared from around the nose, the position with the head lowered, may have added to the drug-induced hypoxemia seen in this study. In moose immobilized with etorphine alone, moose were in sternal recumbency [1,21] with the head raised. Adding xylazine to opioids is not recommended in moose [1] as it results in a deeper immobilization, affecting the positioning (lateral without deep snow, head down) [1,2,22] and resulting in an increased risk of adverse effects including lower hemoglobin oxygen saturation measured by pulse oximetry (J. M. Arnemo, unpublished data) regurgitation and risk of pneumonia [1,22] and higher mortality [23]. Opioids, such as etorphine, produce dose-dependent respiratory depression, primarily by causing the respiratory center of the brain stem to be less responsive to increased PaCO_2_[24]. The responsiveness to PaCO_2_ is further decreased by coadministered sedatives or other anesthetic agents including phenothiazines [24], such as acepromazine. Xylazine which when used alone, causes a dose-dependent decreased responsiveness to CO_2_ that is further compounded when combined with opioids [25]. In sheep, xylazine was shown to cause pulmonary edema resulting in hypoxemia and lung tissue damage [26].

Moderate to severe hypoxemia has also been documented in other ruminants anesthetized with alpha-2 combinations including wood bison (Bison bison) [27], mule deer (*Odocoileus heminus*) [28,29], wapiti (*Cervus canadensis*) [29,30] and white tailed-deer (*Odocoileus virginianus*) [31]. Hypoxemia was also found in cases where alpha-2 combinations were used in combination with opioids [28,30]. The hypoxemia seen in this study as in studies of other ruminants, indicates that oxygen supplementation is indicated in these species. In a study of nine wapiti immobilized with xylazine-tiletamine-zolazepam, all were initially hypoxemic and in all, the hypoxemia resolved after administration of 10 L/minute of nasal oxygen for only five minutes [29]. A study comparing nasal oxygen and medical air supplementation in wapiti before and during anesthesia with carfentanil and xylazine found that wapiti receiving 10 L/minute of oxygen had a significantly faster induction and recovery, less hypoxemia, less rigidity and movement, but more apnea, hypercapnia and acidosis [32]. A review of alpha-2 agonist and hypoxemia concluded that hypoxemia in large ruminants such as cattle are primarily due to hypoventiliation and perfusion mismatching due to recumbency whereas sheep given xylazine can develop pulmonary edema [33]. Evaluation of oxygen flow rates necessary to correct hypoxemia is needed for moose and other species.

The hypercapnia noted indicates hypoventilation, which also causes hypoxemia [34]. Hypercapnia has been documented during anesthesia in a number of ruminants including wood bison [27], mule deer [28,29] and wapiti [29,30]. In a study comparing oxygen and medical air supplementation in wapiti, both groups had increasing PaCO_2_ over time, but this was significantly higher in the group receiving oxygen [32]. Under ordinary conditions, increased PaCO_2_ stimulates and increases central respiratory drive and severely decreased PaO_2_ can also stimulate increased ventilation [35], however anesthetics depress the ventilatory response initiated by increased PaCO_2_. When increasing FiO2 and therefore PaO2 in carfentanil-xylazine immobilised elk a significantly increased PaCO2 was observed which is highly indicative that hypoxemia in the Elk breathing pure air had resulted in increased ventilatory drive [32]. Hypercapnia and decreased pH also cause a right shift in the oxygen-hemoglobin dissociation curve, increasing the unloading of oxygen at tissues, enhancing oxygen delivery [35]. Both etorphine and xylazine are likely contributing to the hypoventilation and intrapulmonary causes of hypoxemia and resulting hypoxemia and hypercapnia. As neither the hypercapnia nor hypoxemia changed with time, this indicates continued respiratory depression.

PAO_2_ are mostly governed by uptake of oxygen by pulmonary capillary blood and replacement by alveolar ventilation. Normal values for PAO_2_ for normal animals at sea level are around 13 kPa [34]. The initial PAO_2_ in this study was 11.3 ± 1.0 (9.8-13.2) kPa. P(A-a)O_2_ was 3.0 ± 1.4 (0.7-5.6) kPa (Normal is generally less than 2 kPa with over 3.3 considered abnormal [35]) indicating that intrapulmonary problems like V/Q mismatch, physiological shunting or diffusion impairment could be contributing factors. Within the study group, we found large variations between animals ranging from normal function to animals with a markedly high P(A-a)O_2_.

The hypoxemia seen in all animals could be due to a variety of possible causes including low inspired O_2_ pressure (altitude), hypoventilation, V/Q mismatch, pulmonary shunting or diffusion limitations. Barometric pressure ranged from 694 to 708 mmHg, so low barometric [35] pressure would not be expected to contribute significantly to the hypoxemia observed. The spread of P(A-a)O_2_ a would indicate most moose have a pulmonary problem in addition to likely hypoventilation. A future evaluation of the effect of positioning (head uphill vs. downhill vs. flat ground) is warranted, however, these large animals can be difficult to reposition beyond moving from lateral to sternal recumbency.

We found a marked acidosis, both respiratory and metabolic. The metabolic part of the acidosis improved with time, reflected in a significantly increased pH and decreased lactate and increased BE in the second sample. The severe lactic-acidemia decreased significantly over time, indicating either decreasing lactate production, increased lactate metabolism or a combination of these. The mean lactate levels in arterial blood in the present study were slightly higher than the mean venous lactate levels in etorphine-immobilized moose reported by [20] who found significantly lower lactic acid levels in animals with longer induction times and with increased time between darting and sampling. That study found a plasma lactate of 9.3 ± 2.1 (2.9-12.5) mmol/L and blood was sampled at 11.0 ± 4.1 (6.0-19.9) minutes after darting. The moose in the current study had both longer induction times, a later sampling time and higher lactate than found in etorphine-immobilized moose [20]. Furthermore, lactate is normally higher in venous samples than in arterial [36]. This indicates that moose immobilized with etorphine-xylazine-acepromazine had higher lactate levels than moose immobilized with etorphine alone.

Anion gap decreased, likely caused by the lactic acidosis. K increased between the samples in spite of an increase in pH. As an increased pH will usually decrease K, the increased K likely reflects that K has leaked out of damaged cells, possibly muscular cells. The decrease in Hb and Hct could be a result of sequestration of erythrocytes in the spleen or increased intravascular fluid volume due to diffusion of interstitial fluid into the vascular space. This usually happens during anaesthesia due to vasodilatation and decreased blood pressure, which is consistent with xylazine and acepromazine anesthesia in some ruminants, however the etorphine usually causes increased blood pressure.

## Conclusions

Moose immobilized with etorphine-xylazine-acepromazine exhibited mild to severe hypoxemia, mild to moderate hypercapnia and moderate to marked acidemia.

Too far reaching comparative conclusions of this study should be cautioned, as it is hard to do cross comparisons among populations as physiological responses to anesthesia might vary in terms of body condition, age distribution and time of the year. The mean induction time for the 12 moose immobilized with one dart in our study was over 2 minutes longer than the 4.4 ± 2.6 minutes reported in etorphine-immobilized moose [20]. Physiological assessment of other immobilizing agents for moose including thiafentanil and etorphine, for anesthesia of moose and evaluation of oxygen supplementation for correction of hypoxemia is warranted.

## Abbreviations

AG: Anion gap; BE: Base excess; Cl: Chloride; HCO_3_: Bicarbonate; PB: Barometric pressure; CO_2_: Carbon dioxide; FiO_2_: Fraction of inspired oxygen (0.21); GPS: Global positioning systems; K: Potassium; Na: Sodium; PaCO_2_: Partial pressure of arterial carbon dioxide; PAO_2_: Alveolar oxygen tension; PaO_2_: Partial pressure of arterial oxygen; P_H2O_: Saturated vapor pressure for water at 37°C (47 mmHg); RQ: Respiratory quotient; SaO_2_%: Percent oxygen hemoglobin saturation.

## Competing interests

The authors declare that they have no competing interests.

## Authors’ contributions

AE participated in study design, carried out the field sampling, data collection and analysis and drafted the manuscript. ÅF participated in design of the study, presentation of data and drafting the manuscript. AH participated in study design and interpretation of results. GE participated in design of the study, statistical analysis and coordinated field logistics. JMA conceived of the study, participated in its design and coordination and helped to draft the manuscript. All authors have critically revised the manuscript and read and approved the final manuscript.

## References

[B1] Arnemo JM Kreeger TJ Soveri T Chemical immobilization of free-ranging moose Alces 2003 39 243 253

[B2] Kreeger TJ Arnemo JM Handbook of wildlife chemical immobilization 4th ed 2012 3 Wheatland, Wyoming: Terry J. Kreeger

[B3] Kreeger T Edwards W Wald E Becker S Brimeyer D Fralick G Berger J Health assessment of Shiras moose immobilized with thiafentanil Alces 2005 41 121 128

[B4] Arnemo JM Ericsson G Oen EO Broman E Heim M Wallin K Os O Ball J Immobilization of free-ranging moose (Alces alces) with etorphine or etorphine-acepromazine-xylazine in Scandinavia 1984–2003: A review of 2,754 captures Proceedings AAZV, AAWV, WDA Joint Conference 2004 2004 515 516

[B5] Delvaux H Courtois R Breton L Patenaude R Relative efficiency of succinylcholine, xylazine, and carfentanil/xylazine mixtures to immobilize free-ranging moose J Wildl Dis 1999 35 38 48 10073344 10.7589/0090-3558-35.1.38

[B6] Seal US Schmitt SM Peterson RO Carfentanil and xylazine for immobilization of moose (Alces alces) on Isle Royale J Wildl Dis 1985 21 48 51 3981744 10.7589/0090-3558-21.1.48

[B7] Cattet M Boulanger J Stenhouse G Powell RA Reynolds-Hogland MJ An evaluation of long-term capture effects in ursids: Implications for wildlife welfare and research J Mammal 2008 89 973 990 10.1644/08-MAMM-A-095.1

[B8] Larsen DG Gauthier DA Effects of capturing pregnant moose and calves on calf survivorship J Wildl Manag 1989 53 564 567 10.2307/3809177

[B9] Neumann W Ericsson G Dettki H Arnemo J Effect of immobilizations on female moose (Alces alces) activity and space use Can J Zool 2011 89 1013 1018 10.1139/z11-076

[B10] Gutierrez G Geoffrey JL, Steven DS Hypoxia and hypoxemia Encyclopedia of respiratory medicine 2006 Oxford: Academic 302 307

[B11] Siesjö B Acidosis and ischemic brain damage Mol Chem Neuropathol 1988 9 31 88 10.1007/BF03160355 3073332

[B12] Wang P Ba ZF H Chaudry I Severe hypoxemia in the absence of blood loss depresses hepatocellular function and up-regulates IL-6 and PGE2 Biochimica et Biophysica Acta (BBA) - Molecular Basis of Disease 1997 1361 42 48 10.1016/S0925-4439(97)00015-X 9247088

[B13] Dyson A Stidwill R Taylor V Singer M Tissue oxygen monitoring in rodent models of shock Am J Physiol Heart Circ Physiol 2007 293 526 533 10.1152/ajpheart.00052.2007 17384135

[B14] Evans RG Goddard D Eppel GA O’Connor PM Factors that render the kidney susceptible to tissue hypoxia in hypoxemia Am J Physiol Regul Integr Comp Physiol 2011 300 R931 R940 10.1152/ajpregu.00552.2010 21248306

[B15] Braaksma MA Dassel ACM Aarnoudse JG Renal responses to prolonged (48 h) hypoxemia without acidemia in the late-gestation ovine fetus Am J Physiol Regul Integr Comp Physiol 1999 277 R395 R402 10.1152/ajpregu.1999.277.2.R395 10444545

[B16] Rees S Breen S Loeliger M McCrabb G Harding R Hypoxemia near mid-gestation has long-term effects on fetal brain development J Neuropathol Exp Neurol 1999 58 932 945 10.1097/00005072-199909000-00004 10499436

[B17] Ericsson GWK Senescence in a northern ungulate: Age and sex-specific patterns of mortality in moose Ecoscience 2001 8 157 163

[B18] Jalanka HH Roeken BO The use of medetomidine, medetomidine-ketamine combinations, and atipamezole in nondomestic mammals: a review J Zoo Anim Med 1990 21 259 282

[B19] Schmidt-Nielsen K Animal physiology: adaptation and environment 1997 Cambridge, England: Cambridge University Press

[B20] Haga HA Wenger S Hvarnes S Os O Rolandsen CM Solberg EJ Plasma lactate concentrations in free-ranging moose (Alces alces) immobilized with etorphine Vet Anaesth Analg 2009 36 555 561 10.1111/j.1467-2995.2009.00498.x 19845927

[B21] Lynch GM Hanson JA Use of etorphine to immobilize moose J Wildl Manag 1981 45 981 985 10.2307/3808108

[B22] Kreeger TJ Xylazine-induced aspiration pneumonia in Shira’s moose Wildl Soc Bull 2000 28 751 753

[B23] Arnemo JM Ahlqvist P Andersen R Berntsen F Ericsson G Odden J Brunberg S Segerstrom P Swenson JE Risk of capture-related mortality in large free-ranging mammals: experiences from Scandinavia Wildl Biol 2006 12 109 113 10.2981/0909-6396(2006)12[109:ROCMIL]2.0.CO;2

[B24] Gustein HB Akil H Brunton LL Opioid analgesics Goodman & Gilman’s the pharmacological basis of therapeutics 2006 11 New York: McGraw/Hill 560 568

[B25] Grimm KA Lamont L West G, Heard D, Caulket N Clinical pharmacology Zoo animal and wildlife immobilization and anesthesi 2008 1 Ames, Iowa, USA: Wiley-Blackwell 3 36

[B26] Celly CS Atwal OS McDonell WN Black WD Histopathologic alterations induced in the lungs of sheep by use of alpha(2)-adrenergic receptor agonists Am J Vet Res 1999 60 154 161 10048544

[B27] Caulkett NA Cattet MR Cantwell S Cool N Olsen W Anesthesia of wood bison with medetomidine-zolazepam/tiletamine and xylazine-zolazepam/tiletamine combinations Can Vet J 2000 41 49 53 10642872 10.4141/cjas61-007 PMC1476335

[B28] Caulkett NA Cribb PH Haigh JC Comparative cardiopulmonary effects of carfentanil-xylazine and medetomidine-ketamine used for Immobilization of mule deer and mule deer/white-tailed deer hybrids Can J Vet Res 2000 64 64 68 10680659 PMC1189583

[B29] Read M Caulkett NA Symington A Shury TK Treatment of hypoxemia during xylazine-tiletamine-zolazepam immobilization of wapiti Can Vet J 2001 42 861 864 11708204 PMC1476651

[B30] Storms TN Schumacher J Zagaya N Osborn DA Miller KV Ramsay EC Determination and evaluation of an optimal dosage of carfentanil and xylazine for the immobilization of white-tailed deer (Odocoileus virginianus) J Wildl Dis 2005 41 559 568 16244066 10.7589/0090-3558-41.3.559

[B31] Siegal-Willott J Citino SB Wade S Elder L Hayek LA Lance WR Butorphanol, azaperone, and medetomidine anesthesia in free-ranging white-tailed deer (Odocoileus virginianus) using radiotransmitter darts J Wildl Dis 2009 45 468 480 19395756 10.7589/0090-3558-45.2.468

[B32] Paterson JM Caulkett NA Woodbury MR Physiologic effects of nasal oxygen or medical air administered prior to and during carfentanil-xylazine anesthesia in North American elk (Cervus canadensis manitobensis) J Zoo Wildl Med 2009 40 39 50 10.1638/2007-0107.1 19368239

[B33] Read MR A review of alpha-2 adrenoreceptor agonists and the development of hypoxemia in domestic and wild ruminants J Zoo Wildl Med 2003 34 134 138 12885129 10.1638/1042-7260(2003)034[0134:AROAAA]2.0.CO;2

[B34] West JB Respiratory Physiology 2008 8 Hong Kong: Lipincott Williams & Wilkins 186

[B35] Johnson RA Morais HA DiBartola SP Respiratory acid–base disorders Fluid, electrolyte and acid–base disorders 2011 St. Louis, Missouri: Elsevier Inc 287 301

[B36] Forster HV Dempsey JA Thomson J Vidruk E DoPico GA Estimation of arterial PO2, PCO2, pH, and lactate from arterialized venous blood J Appl Physiol 1972 32 134 137 5007005 10.1152/jappl.1972.32.1.134

